# Does the trajectory of NLR fall short of its absolute value in predicting prognosis in acute heart failure?

**DOI:** 10.1093/eschf/xvag071

**Published:** 2026-03-05

**Authors:** Akinori Higaki

**Affiliations:** Department of Cardiology, Pulmonology, Hypertension & Nephrology, Ehime University Graduate School of Medicine, 454 Shitsukawa, Toon 791-0295, Japan

**Keywords:** Neutrophils-to-lymphocytes ratio, Acute heart failure, Prognosis

To the Editor,

I read with great interest the recent study by Marcus *et al*.^1^ examining the association between the neutrophil-to-lymphocyte ratio (NLR) and prognosis in patients hospitalized with acute decompensated heart failure. In their analysis of more than 6000 patients, the authors demonstrated that discharge NLR predicted 30-day outcomes more accurately than admission NLR. We previously reported, in patients with COVID-19 who had cardiovascular disease or its risk factors, that NLR measured immediately before discharge was more strongly associated with mortality than NLR at admission.^2^ In this respect, the findings of Marcus et al. appear consistent with our observations. Notably, the authors went a step further by focusing on the temporal change in NLR—its trajectory—from admission to discharge. Using a cut-off value of 5, they categorized patients into four groups and showed that the high-to-high group (NLR ≥5 at both admission and discharge) had the highest mortality among the four categories. Interestingly, however, the 30-day readmission rate was slightly worse in the low-to-high group than in the high-to-high group. This study provides valuable insight by incorporating a temporal dimension into the relationship between inflammatory status and prognosis in heart failure. Nevertheless, the use of a single cut-off value may oversimplify the data and potentially obscure clinically meaningful information.

For example, a patient whose NLR decreases from 10 at admission to 6 at discharge would be classified in the high-to-high group, despite apparent improvement in inflammatory status. In contrast, a patient whose NLR increases from 3 to 6 would be categorized as low-to-high, reflecting a worsening inflammatory profile. Intuitively, the latter scenario—rising inflammation despite heart failure treatment—may suggest suboptimal response and possibly worse prognosis. Although both patients share the same discharge NLR, the current analysis does not clarify whether their outcomes differ. Moreover, the mean discharge NLR values for each of the four trajectory groups were not presented, making it difficult to determine whether the poorer prognosis observed in the high-to-high group simply reflects a higher absolute discharge NLR compared with the low-to-high group. If so, the incremental prognostic value of admission NLR may be limited. Conversely, if the magnitude of change in NLR (delta NLR) is independently associated with outcomes, this would further support the clinical relevance of serial NLR assessment, as previously demonstrated by Boralkar *et al*.^3^

As acknowledged by the authors, data on B-type natriuretic peptide—a key biomarker in heart failure—were unavailable. Given this limitation, many readers may be particularly interested in a more granular analysis of the available NLR data. For example, a scatter plot with admission NLR on the x-axis and discharge NLR on the y-axis, with data points colour-coded according to subsequent cardiac events, might provide additional insight into the prognostic implications of NLR dynamics. I hope that Marcus et al. will further explore their valuable dataset to provide deeper insight into the inflammatory trajectories associated with heart failure outcomes.

## Declarations

### Data Availability

No data were generated or analysed for this manuscript.

## References

[1] Marcus G, Maymon SL, Kalmanovich E, Moravsky G, Minha I, Grupper A, et al (February 19, 2026) Discharge neutrophil-to-lymphocyte ratio and its trajectory as predictors of 30-day outcomes in acute heart failure. ESC Heart Fail, 10.1093/eschf/xvag059 PMC13122620 41711242

[2] Higaki A, Okayama H, Homma Y, Sano T, Kitai T, Yonetsu T, et al Predictive value of neutrophil-to-lymphocyte ratio for the fatality of COVID-19 patients complicated with cardiovascular diseases and/or risk factors. Sci Rep 2022;12:13606. 10.1038/s41598-022-17567-4 35948607 PMC9364304

[3] Boralkar KA, Kobayashi Y, Amsallem M, Ataam JA, Moneghetti KJ, Cauwenberghs N, et al Value of neutrophil to lymphocyte ratio and its trajectory in patients hospitalized with acute heart failure and preserved ejection fraction. Am J Cardiol 2020;125:229–35. 10.1016/j.amjcard.2019.10.020 31753313

